# Supplementary material for: Chromothripsis during telomere crisis is independent of NHEJ, and consistent with a replicative origin
Source: Genome Res. 2019 May;29(5):737–49. doi: 10.1101/gr.240705.118 (PMC6499312; doi:10.1101/gr.240705.118)
Supplement: Supplemental Material [file supp_gr.240705.118_Supplemental_file_1.zip › contigs/annotated_contigs/DB55/contig.3.DB55_length_405_mean_cov_6.12098765432.docx]

**DB55_length_405_mean_cov_6.12098765432**

```
AAAGAAGAAGAAGAACTTAAAAAAAATCCTTTAATATCCATTAGTTAAAATTTTAAAAGGCTAGAGTGGT|AGGCGCAGTGGCT|TATG
 >chr1:44650555-44650638 - E=2e-35 p=0e+00                             >chr7:144830738-
CCTGTAACCCCAGCGTTTTGGGAGGCCGAGGCGGGCGGATCACTTGAGATCAGGAGTTCAAGACCAGCCTGGCCAACATGCTGAAACCC
144830879 - E=2e-73 p=4e-02
CATCTCTACTAAAAATACAAAAAAATTAACTGGGT|ATTAA|CTGGAACTAAGGTTCTGGCTTTTGAATTCATTGGTATTTTGAGAGCA
                                         >chr7:144373988-144374177 - E=3e-93
ATATGTACCATGAATATCAGTGCTAAATGTGAGGTTTTTTTTTTATTTGTGATTTTGTGGTACTTAGAGATTCTCAGGCTTATTAGCAT

AACAGTCTAAGAAGTTGCTATATCTTTAATATTGTGTGTAGGATATCTGAAAT
```
